# A feasibility RCT protocol of the MATILDA intervention to support older adults with intellectual disability to improve their health and social networks

**DOI:** 10.1186/s40814-026-01780-3

**Published:** 2026-02-18

**Authors:** Laurence Taggart, Angela Hassiotis, Assumpta Ryan, Brendan Bunting, Carian O’Neill, Mike Clarke, Roger Stancliffe, Haleemah Ahmed, Elle Kim, Lindsay Armstrong, Andrew Hanna, Susie Willis, Brendan Leahy, Janet Schofield, Allison Campbell

**Affiliations:** 1 School of Nursing & Midwifery, Queens University Belfast, Lisburn Road, Belfast, BT7 INN Northern Ireland; 2 Institute of Psychiatry, University College London, London, England; 3 School of Nursing and Paramedic Science, Ulster University, Derry, Northern Ireland; 4 N Ireland Clinical Trials Unit, Belfast, Northern Ireland; 5 Centre for Disability and Research, Sydney University, Sydney, Australia; 6 Volunteer Now, Belfast, Northern Ireland; 7 Camden & Islington NHS Foundation, London, England; 8 Greenwood Services, Camden & Islington, London, England; 9 CAN Compass Advocacy Network, Ballymoney, Northern Ireland

**Keywords:** Intellectual disability, Older adults, Community groups, Feasibility RCT study, Normalization Process Theory

## Abstract

**Background:**

Older adults with an intellectual disability are at greater risk of increased social isolation, loneliness, and poorer quality of life compared to their non-disabled ageing peers. The MATILDA study aims to improve health and social networks by facilitating older adults with an intellectual disability to engage in their local mainstream community group.

**Methods:**

The 30-month project is a 2-arm, single-blind, randomised feasibility RCT with 1:1 allocation, being conducted in Northern Ireland and London, England. We are aiming to recruit 64 older adults with an intellectual disability (aged 45 plus) who will be randomised to either the MATILDA intervention or an active control group alongside usual care. We will assess feasibility outcomes (i.e. recruitment, acceptability of intervention, retention, etc.). We will also conduct a process evaluation using the Normalisation Process Theory to identify any solutions and challenges in implementing the MATILDA intervention in local community groups.

**Discussion:**

This is a novel, naturalistic experiment, low-cost community intervention that supports older adults with intellectual disability to engage in local community groups. This study builds upon our earlier work in exploring how older adults with intellectual disability age, engage in local community activities, and what are the enablers and barriers to facilitators in joining local community groups.

**Trial registration:**

ISRCTN, ISRCTN15294181. Registered 20th December 2022.

**Supplementary Information:**

The online version contains supplementary material available at 10.1186/s40814-026-01780-3.

## Background

People with an intellectual disability (ID) are living significantly longer than in previous decades, but they experience ageing earlier and at a faster rate than their non-disabled peers [1]. This ageing ID population is predicted to grow four times faster than the general population, placing increasing pressure on health and social care systems [2]. Despite this demographic shift, the needs of older adults with an ID remain underspecified and under-researched.

Unlike non-disabled older adults, chronological age, retirement, and access to pensions do not typically define ageing for adults with an ID [3, 4]. Many have not engaged in paid employment and therefore lack the structural transition points or social markers that signify ageing in the general population [5, 6]. Consequently, they often have limited opportunities to participate in community life alongside their non-disabled peers [7]. This exclusion contributes to loneliness, isolation, and depression, yet neither intellectual disability services nor mainstream ageing services have adequately addressed how to support inclusion for this population [2, 8–13]. Recent UK policy emphasises the importance of community inclusion and social participation for people with disabilities, including the NICE Guidelines (NG96), which recommend ensuring that older adults with an ID have equal access to mainstream services and social opportunities [14–17]. However, the mechanisms by which this can be achieved in practice remain unclear.

### Older adults with an intellectual disability and the impact of loneliness/isolation

Loneliness and social isolation are well-established determinants of poor physical and mental health in the general ageing population [18, 19]. Older adults with an ID face even greater risks due to limited social networks, communication challenges, and negative societal attitudes [8, 9, 20–23]. Petroutsou et al. reported that over half (50.4%) of adults with an ID experience loneliness compared with 10.6% of the general population [21]. This is despite many expressing a desire to remain active and socially connected [20].

Improving social connectedness has proven benefits: meta-analyses in older populations show that interventions enhancing social skills, increasing social support, and creating opportunities for engagement can significantly reduce loneliness [24–26]. Yet, how to translate these approaches to meet the needs of older adults with an ID remains poorly understood.

### Existing approaches: befriending schemes

Befriending schemes have been widely used to address loneliness and isolation, typically involving a volunteer who provides one-to-one social support and companionship. Such schemes can improve perceived social support and psychological wellbeing [27] and may also benefit volunteers through enhanced empathy and community cohesion [28].

However, their effectiveness for people with an ID is uncertain. Small feasibility studies have shown mixed results: Florides [29] reported improved confidence and reduced isolation in a small London-based pilot, while Ali et al. [30] found that despite positive experiences, recruitment challenges limited feasibility. Furthermore, traditional befriending models often rely on short-term, prescriptive relationships that may end abruptly, leaving participants feeling distressed and socially adrift [31, 32]. These schemes tend to provide companionship rather than facilitating long-term inclusion within community networks, and thus do little to address the structural exclusion faced by older adults with an ID.

### Community-based mentoring: the Australian TTR project

The Transition to Retirement (TTR) project, developed in Australia, represents a more embedded, community-based approach [33]. It used active mentoring to support older adults with an ID (aged ≥45) to join existing mainstream community groups. Retired community members acted as mentors, helping individuals with an ID to participate in activities such as bowling, gardening, or crafts. Participants attended groups one to three times per week over 6 months, supported by two to three mentors per participant.

Results were promising: participants reported reduced loneliness, improved wellbeing, and increased social networks. Importantly, many continued attending community groups independently after the study ended, suggesting that community integration was sustainable. The TTR approach thus moved beyond the one-to-one befriending model by focusing on facilitated inclusion within existing community structures.

Nevertheless, the TTR study had limitations. It targeted adults transitioning from supported employment, limiting applicability to the broader group of older adults with an ID who are not in work. The intervention was also implemented within the Australian service context, which differs considerably from the UK in terms of community infrastructure, volunteer culture, and disability services.

### The MATILDA intervention

Building on the TTR model, our team adapted and expanded it for a UK context, developing the MATILDA intervention (Managing Activities Together to Involve older adults with a Learning Disability in their Local Areas). MATILDA maintains the core principle of enabling older adults with an ID to participate in local community groups but introduces several key innovations:

1. Broader inclusion — MATILDA targets all older adults with an ID, not only those transitioning from employment.
2. Mentor pairing model — Each participant is supported by one or two trained community mentors, rather than a single befriender. This reduces pressure on volunteers and fosters more natural network development within the group.
3. Group integration focus — Mentors work within existing community groups (e.g. walking clubs, arts groups) to promote acceptance and inclusion of the person with an ID.
4. Sustainability — By embedding social connections in mainstream settings, MATILDA aims to create lasting friendships and reduce dependency on structured disability services.

In contrast to befriending schemes, which often provide temporary companionship, MATILDA focuses on social integration, helping people with an ID to belong and participate meaningfully in community life. The dual-mentor model enhances support, while community group involvement increases opportunities for naturally sustained relationships. This approach also reduces reliance on costly formal support and leverages existing social infrastructures, aligning with policy goals for inclusive, community-based care.

### Rationale for a feasibility RCT

Given the promising theoretical and preliminary evidence, it is now timely to conduct a feasibility randomised controlled trial (RCT) of MATILDA. This study will test the practicality and acceptability of conducting a full-scale trial to evaluate the intervention’s effects on social connectedness, wellbeing, and quality of life.

Key feasibility questions include:

- Can older adults with an ID be successfully identified, recruited, and consented through existing service and community pathways?
- Is the MATILDA intervention acceptable to participants, mentors, and community groups?
- Can mentoring relationships and community participation be sustained over the study period?
- Are proposed outcome measures and data collection procedures appropriate and sensitive to change?

The feasibility RCT will also assess the logistics of randomisation, mentor matching, and retention, providing essential data to inform the design and power calculation of a definitive trial. If feasible, MATILDA could offer a scalable, low-cost, and sustainable approach for commissioners and service providers. By using existing community assets and volunteer capacity, it aligns with national strategies to build inclusive, age-friendly communities [32, 34] and to reduce loneliness among people with disabilities. It would also contribute to UK’s broader policy ambitions around personalisation, prevention, and community participation in social care.

Older adults with an intellectual disability represent a growing yet underserved population at high risk of social isolation and poor wellbeing. Traditional befriending approaches, while valuable, are limited by short-term, one-to-one structures that fail to create sustainable community integration. The Australian TTR model demonstrated that active mentoring can effectively embed older adults with an ID in community groups, but adaptation for the UK context is essential.

MATILDA represents an innovative evolution of this model: a community-based, mentor-supported pathway that promotes long-term social inclusion, wellbeing, and sustainability. This feasibility RCT will establish the groundwork for a future definitive trial and, ultimately, for a scalable intervention capable of improving lives while strengthening inclusive communities.

### Aim and objectives of study

The primary aim of this study is to conduct a UK based, multicentre, feasibility RCT to determine the feasibility (i.e. recruitment, consent, matching, retention, dropout etc.), and acceptability of the Matilda intervention for adults with an intellectual disability targeting health, wellbeing and social networks compared to usual care.

We have planned to assess several secondary outcomes including clinical outcomes (i.e. quality of life, anxiety, loneliness) to:

1. Explore the views of the stakeholders (adults with an intellectual disability, carers, and mentors) about the acceptability of the Matilda intervention
2. Determine the appropriateness and acceptability of the outcome measures to older adults with an intellectual disability, carers, and mentors
3. Measure the fidelity of mentors in supporting older adults with an intellectual disability
4. Estimate the effect of the Matilda intervention on the outcomes for the older adults with an intellectual disability (health, wellbeing, and social connectedness), family carers (health, wellbeing), and for the mentors (wellbeing, attitudes to people with an intellectual disability) at 6 and 12 months post intervention
5. Provide preliminary information about treatment effects to inform the sample size for a full trial
6. Record any adverse events and unintended consequences of the Matilda intervention

## Methods

### Study design

**Fig. 1:**
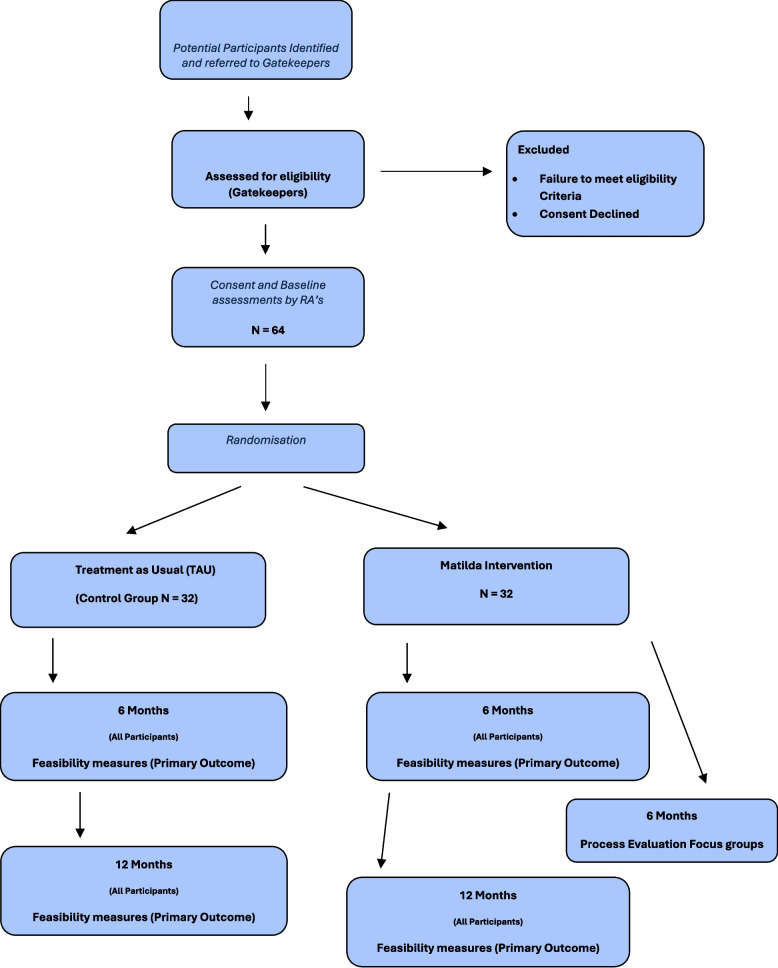
Study schematic diagram

This is a 2-arm, single-blind, randomised feasibility study with 1:1 allocation, which will be conducted in Northern Ireland and London (Fig. 1). Sixty-four older adults with an intellectual disability will be randomly allocated to either the Matilda intervention plus usual care (‘usual care’ in this instance refers to participants attending a day centre or day opportunity setting) or usual care with three group recreational activities with other older adults with an intellectual disability (active control arm). Group recreational activities will involve older adults with an intellectual disability in the active control arm meeting together on three occasions for example for a meal, a coffee or bowling, etc.

The intervention will last 6 months. The primary outcome is feasibility (i.e. recruitment, consent, matching, retention, dropout, etc.). Outcome measures will be collated at baseline, 6 months post intervention and 12 months post intervention. We will conduct a process evaluation and a health economic evaluation.

The feasibility trial will run for 30 months (to include start up right through to project close down) to assess recruitment rates and retention for the measuring of the primary outcome at 6 months post intervention and 12 months intervention. This will also allow us to identify any key difficulties and address them in preparation for a potential definitive randomised trial. The two clinical sites, one in Northern Ireland and one in London, will recruit a total of 64 older adults with an intellectual disability during this period. This protocol is reported in accordance with the Standard Protocol Items: Recommendations for Interventional Trials (SPIRIT) guidelines.

### Ethical approval

Ethical approval was received by the Office for Research Ethics Northern 249 Ireland (ORECNI: 22/NI/0067), and research governance was obtained from both sites in Northern Ireland and England.

### Progression criteria

We will apply predetermined progression criteria as set by Avery et al. [34]. This will be based on the feasibility outcome data pertaining to recruitment, matching older adults with an intellectual disability to mentors, retention rates and reasons for attrition, programme attendance, protocol adherence, and the completion of outcome measures. We will monitor recruitment rates in each site. We will have a wide recruitment network from the outset including user-friendly information on how to promote the study and meetings with various local community organisations. We will identify champions in both sites to help study promotion and a process evaluation will take place with stakeholders.

A traffic light system (green, amber, red) will be used to inform whether the study should progress to a full trial, whether revisions are required, or whether it should be stopped. If there is efficacy, and the green progression criteria are met, it would be appropriate to move to a definitive trial, see Table 1.

**Table 1:** Progression criteria: a traffic light system

Progression criteria	Red	Amber	Green
1) Recruitment rates of adults with an intellectual disability and mentors who are approached consenting to randomisation across both sites	<39%	40–69%	70% plus
2) Acceptability of and engagement in the Matilda intervention of at least 60% of the adults with an intellectual disability allocated to this arm across both sites	<39%	40–59%)	>60%
3) Acceptability of the matching process, and engagement in the Matilda intervention of at least two mentors for at least 60% allocated to this arm across both sites	<39%	40–59%)	>60%
4) Acceptability of the appropriateness of the outcome measures for at least 60% of the adults with an intellectual disability and their mentors across both sites	<39%	40–59%)	>60%
5) Retention of at least 60% of the recruited adults with an intellectual disability and their mentors at the 6- and 12-months follow-up across both sites	<39%	40–59%)	>60%

### Setting

The study will take place in one region of Northern Ireland, the Northern Health and Social Care Trust, and in one region of London, Camden and Islington. We have chosen these sites to maximize recruitment across a large population and improve the applicability of our results. They cover a range of urban and semi-rural areas and include areas with high levels of deprivation (a lack of resources, opportunities and/or services) and where London’s population is ethnically and culturally diverse. We will use a range of mainstream community older persons’ groups to deliver the Matilda intervention accessed via Volunteer Now in Northern Ireland and the Camden & Islington Foundation Trust volunteer service in London.

To be involved in the study, the older person, mainstream community groups, and mentors at the clinical sites must be prepared to participate in the Matilda training and be prepared to support an older person with an intellectual disability in the group. They must also demonstrate and document a willingness to comply with the study protocol and applicable regulatory requirements, sponsor, and local policies.

### Participants — older adults with an intellectual disability

Older adults with an intellectual disability (≥45 years) living in the community will be assessed using the inclusion and exclusion criteria as set out below. According to the American Association on Intellectual and Developmental Disabilities (AAIDD), intellectual disability is characterized by significant limitations in both intellectual functioning (reasoning, learning, problem solving) and in adaptive behavior, which covers a range of everyday social and practical skills, and originates before the age of 18 [35]. Eligibility to participate in the trial will be confirmed by a health and social care provider.

### Inclusion criteria

Adults with an intellectual disability will be eligible to participate in the study in accordance with the following criteria:

- Mild/moderate intellectual disability
- Living in the community with a family member(s) or in any type of community accommodation (residential/supported)
-  ≥45 years
- Able to communicate verbally and
- Able to provide informed consent.

### Exclusion criteria

Adults with an intellectual disability will be excluded to participate in the study in accordance with the following criteria:

- Severe/profound intellectual disability
- Presenting with severe challenging behaviour
- Unable to communicate verbally or in English
- Unable to provide consent and
- Already accessing mainstream community groups.

### Sample size

The chosen sample size is justified based on feasibility outcomes, specifically estimating the proportion of adults with intellectual disability who are recruited and retained. With 64 participants, if observed retention is 60%, the 95% CI for the true proportion retained would be approximately 46% to 71%.

### Participants and mentors

#### Screening and recruitment strategy

Posters and flyers will be used to recruit potential local community groups and mentors in each site. The campaign will be run by the mainstream Volunteer Now organisation in Northern Ireland and Camden & Islington Foundation Trust volunteer service in London. A Volunteer Co-ordinator will be appointed to each site and based in each of these organisations. The Volunteer Co-ordinator will provide information and awareness-raising sessions to those local community groups interested in the aim and purpose of the Matilda project; answering any questions the groups may have and meeting potential mentors. People interested in being mentors in the study will be asked to complete an application form to ensure they meet the inclusion criteria and attend an informal interview with the Volunteer Co-ordinator. When written consent has been obtained from those meeting the above criteria to participate in the study as a mentor, the research staff will arrange to undertake the baseline questionnaires within 2 weeks.

### Inclusion criteria

People will be eligible to participate in the study as a mentor in accordance with the following criteria:

- Attend a local community group
- Provide written consent
- Complete registration and declaration of convictions forms

### Exclusion criteria

People will be excluded from participation in the study as a mentor in accordance with the following criteria:

- Have a criminal record
- Consent declined

### Training mentors

Mentor training will be delivered by the Volunteer Co-ordinator in each site and will cover background to the Matilda project and the role of the mentor, disability awareness training, working with adults with intellectual disability, communication strategies, and safeguarding training (incl. confidentially, identifying signs of abuse, managing behaviours that challenge, lone working). This group training will last 3 h, and mentors will be provided with tea/coffee and lunch. The mentors will also receive monthly supervision from the Volunteer Co-ordinator in each site, either face-to-face, by telephone, or online to ensure the safety/wellbeing of the adult with an intellectual disability and check the fidelity of the intervention.

In Northern Ireland, we will work very closely with Volunteer Now. Volunteer Now is the lead organisation for promoting and supporting volunteering across Northern Ireland. It supports local community groups and voluntary organisations through the provision of training, promoting their volunteering opportunities, support with safeguarding, etc. Volunteer Now supports more than 40 mainstream older people community groups across Northern Ireland such as Men’s Shed, Knit and Knatter, recreational groups (i.e. bowling), etc. They have access to approximately 3000 older adults without an intellectual disability who are registered volunteers who could be potential mentors for the Matilda project. Volunteer Now will recruit the Volunteer Co-ordinator, identify, and recruit the local community groups and the mentors, and provide the training and supervision for the mentors for the course of the Matilda project. Volunteer Now will work closely with the research team and the Volunteer Co-ordinator in the Camden & Islington Foundation Trust Volunteer Service, London.

In London, we will work with the Camden & Islington Foundation Trust volunteer service who will advertise and recruit the Volunteer Co-ordinator, identify the local community groups and mentors, and provide the training and supervision of the mentors. Part of the Camden & Islington Foundation Trust Volunteer Service includes a section for Activity Support and Befriending Volunteers (Services for Ageing Mental Health). The leader of the Camden & Islington Foundation Trust Volunteer Service will work closely with Volunteer Now to ensure the uniformity of the training, monitoring, and supervisory arrangements across the two sites.

### Consent

Written informed consent must be obtained by the Research Associate (RA) on each site prior to the collection of trial data and the provision of the intervention. It must be assumed that an adult with an intellectual disability has the capacity until proven otherwise. Therefore, it is important that the person is given the information required in a user-friendly format to make an informed decision using reasonable adjustments. Managing on-going consent, including assessment of capacity to consent, will take place using the following steps.

Research Associates will undertake the Good Clinical Practice Course and receive specific training at each site in how to assess capacity to consent and ensure informed consent is maintained throughout the project on a case-by-case basis. All participants will receive an easy read Participant Information Sheet (PIS) and consent form with pictures or symbols to explain the purpose of the study and what is involved. The PIS and consent form have been prepared in collaboration with our PPI representatives. The RAs will clearly explain the decisions to be made about joining the Matilda Project, completing some questionnaires at several time points, being randomised to either the intervention or control arm, and taking part in the process evaluation interview (qualitative study) using the user-friendly PIS or additional media or communication aids if required. The Research Associates will explain what is involved in participating in the Matilda Intervention (joining a community group, being matched to two or three mentors within the group, time commitments) and being randomised to the control group. The Research Associates will assess if the person can retain the information and where appropriate have present a family member or advocate who is familiar with the communication needs of the person with the intellectual disability.

### Matilda intervention

The original TTR social intervention was established to explore the retirement options for older adults with an intellectual disability engaged in employment in Australia and to assess possible pathways to retirement [36]. The theoretical underpinnings of the TTR intervention are the Active Support Model [37] and the Co-Worker Training Model, where potential mentors are provided with training and then matched with an older adult with an intellectual disability to access a local community group: it is a peer-led intervention.

The TTR intervention has three components: (1) promoting the concept of retirement, (2) laying the groundwork for inclusion of would‐be retirees with an intellectual disability, and (3) constructing the reality. The third component comprises five stages: planning, locating a group, mapping new routine, recruiting and training mentors, and monitoring and ongoing support [37]. Adults with an intellectual disability reported increased social connectedness, less loneliness, improved wellbeing, and better quality of life after engaging in the TTR project. No data were collected on the benefits of the project for the mentors. In consultation, it was agreed that this intervention could be adapted to older people with an intellectual disability generally, regardless of employment status.

We adapted the TTR project to a UK context. We held 7 focus groups with senior staff from local disability and mainstream older person charities, potential mentors, older adults with an intellectual disability, and their carers. Three co-production workshops with key stakeholders explored how the TTR project could be adapted for a UK context. The key issues identified were agreement to give less emphasis to retirement, reflected in its new acronym ‘Matilda’.

### Matching of adults with an intellectual disability, mentors, and local community groups

The Volunteer Co-Ordinator and the disability staff in each site will meet with one older adult with an intellectual disability and their carers at a time, to explore the person’s needs, capabilities, interests, and hobbies, to identify a suitable local community group and discuss the logistics of attendance (availability of both the adult with the disability and the mentors, transportation to and from the community group, when and where the older adults and mentors would meet, etc.).

The Volunteer Co-ordinator will support the 1–2 mentors to facilitate the person with the intellectual disability to attend the local community group. This will continue until the mentors feel they are confident in supporting the adult with the intellectual disability, and the adult is comfortable with the arrangements. Support will be individually tailored, with the Volunteer Co-ordinator regularly following up to ensure that the adult with the intellectual disability is attending and actively involved in the community group (visits, phone, text). Mentors and carers will be able to contact the Volunteer Co-ordinator and intellectual disability staff for advice. By their nature, local community groups such as men’s sheds, sporting activities, knitting, or gardening groups vary in their frequency and duration, typically between 1 and 4 h over 1 to 3 sessions per week. The adult with the intellectual disability will be supported by their mentors to attend the group and facilitate their involvement with the group for 6 months.

### Control or comparator group

Participants randomised to the Matilda group will also receive usual care, ensuring that they do not lose any care that is standard. Those in the control group will receive usual care and will be offered three group recreational activities. Details regarding participants’ usual care will be confirmed at the start of the study and again at the end of the study. The participants in the control group will complete data gathering instruments at baseline, 6, and 12 months from consent being given.

### Randomisation and assignment of interventions

Following informed consent, older adults with an intellectual disability will be randomised using a 1:1 ratio to the intervention group (Matilda) or control group, stratified by site. The randomisation will be generated by a member of staff based at the Northern Ireland Clinical Trials Unit not connected to the study. The research staff at Ulster University and University College London will inform the adults with an intellectual disability which arm they have been allocated to. At the time of randomisation, each participant will be allocated a unique Participant Study Number, which will be used throughout the study for participant identification. The research staff conducting the baseline and follow-up assessments will not be blinded to each participant’s allocated group. It is not possible to blind the adults with an intellectual disability and the mentors to arm allocation.

### Outcome measures

#### Primary outcome measure

We will use both quantitative and qualitative methodologies to examine the feasibility outcomes of the Matilda intervention (i.e. assess eligibility, recruitment rates and pathways, consenting rate, randomisation process, matching of mentors (and local community groups) and older adults with an intellectual disability, training and supervision, attendance levels, dropout rates, and retention of participants).

#### Secondary outcome measures for adults with intellectual disability

These have been chosen based on the measures used in the original TTR project and our experience with older adults with intellectual disability. They reflect the total measurement load that adults with an intellectual disability have been willing to bear, and the need for brevity and good psychometric properties.

- Health-related QOL**:** WHOQOL-Dis is a measure of health-related quality of life developed by the WHO [38]. This 26-item short version consists of two benchmark items on general health (not used in the scoring) (one general QOL item, one general health item) and 24 specific items which generate a total score and four domains: physical health, psychological health, social relationships, and environmental QOL. Each item is answered on a 5-point Likert scale, which assesses the intensity, capacity, frequency, and evaluation of QOL facets with respect to the last 2 weeks. The WHOQOL-Dis has been validated in intellectual disability individuals.
- Anxiety: Glasgow Anxiety Scale for people with Intellectual Disability (GAS-ID) was developed for adults with an intellectual disability [39], which is a 27-item self-report scale. It has been used in several psycho-interventions for adults with an intellectual disability and found to have good psychometric properties. Scores range from 0 to 40, and higher scores indicate more symptoms.
- Loneliness and social satisfaction: The Modified Worker Loneliness Scale is a 12-item questionnaire that measures loneliness in adults with an intellectual disability. It has been used in several intellectual disability interventions and found to have good psychometric properties [40].
- WHO Wellbeing 5**:** A short 5-item scale to measure subjective wellbeing [38].
- Service use and costs: The study-specific Client Receipt Services Inventory [41] will be used to collate service use and costs for people with an intellectual disability over the 12 months of the study for the preceding 6 months at each follow-up point. It will be completed by the adult with an intellectual disability and their family/paid carer.
- EQ-5D-Y [42]: This questionnaire has simplified wording and has been used previously in adults with an intellectual disability (Jahoda et al., 2017). It is a generic preference-based measure of health-related quality of life, which provides a description of health using five dimensions (mobility, self-care, usual activities, pain/discomfort, and anxiety/depression), each with levels of severity.

#### Secondary outcome measures for mentors

- Health Related Quality of Life: WHOQOL-Dis is a measure of quality of life developed by the WHO [43]. This 26-item short version consists of four domains (physical health, psychological health, social relationships, and environment QOL).
- Psychological well-being and quality of life: The Warwick Edinburgh mental wellbeing scale (WEMWBS) [44] is a 14-item questionnaire that measures psychological well-being and quality of life in the adult general population.
- Attitudes Towards People with an intellectual disability: The 67-item Attitudes Towards Intellectual Disability Questionnaire (cognitive, affective, and behavioural components) [45].
- EQ-5D-Y [46]: This is a generic preference-based measure of health-related quality of life, which provides a description of health using five dimensions (mobility, self-care, usual activities, pain/discomfort, and anxiety/depression), each with levels of severity.

As a token of gratitude and to encourage completion of the measures, the adults with an intellectual disability and their mentors will each be offered a financial incentive of a £10 voucher at each of the three time points. A £10 payment will be given to each adult with an intellectual disability in the intervention arm to recompense them for their time spent on completing the research measures at each of the three time points (not the intervention itself). Similarly, a £10 payment will be given to each adult with an intellectual disability in the control arm to recompense them for their time spent on completing the research measures at each of the three time points — baseline, 6 months post intervention, and 12 months post intervention.

### Process evaluation

The process evaluation will examine four key aspects of the feasibility of conducting a definitive trial of the Matilda intervention: (1) intervention recruitment, adherence, and reach, (2) intervention implementation, (3) intervention mechanisms, including receipt and acceptability, and (4) the feasibility of implementing Matilda within a definitive randomised trial [46]. This will employ a convergent mixed methods approach:

- Recruitment, matching, adherence (how often the adults with an intellectual disability and mentors meet weekly over the 6 months) and reach will be recorded by the mentors using the weekly logs. This will be further explored during the focus groups with the participants in the process evaluation.
- Intervention implementation and modifications will be recorded through the focus groups with the mentors exploring their engagement with the adults with an intellectual disability and key influences on implementation (perceptions of the relationship; barriers/enablers to implementation).
- Intervention mechanisms, receipt and acceptability needed (incl. benefits and/or adverse effects or unintended consequences) will be recorded through the focus groups with all participants, as well as with the disability staff and Volunteer Co-ordinators. We will explore recruitment, acceptability of randomisation, appropriateness of the outcome measures, acceptability, and engagement of the adults with an intellectual disability and mentors in the groups, and reasons for not engaging/dropout.

The above data (on recruitment, intervention implementation, and intervention mechanisms) will help inform the assessment of the feasibility of implementing and willingness to participate in a later definitive randomised trial.

We will also conduct a process evaluation using the Normalisation Process Theory to identify any solutions and challenges in implementing the MATILDA intervention in local community groups. We will hold focus groups with staff to support the adults with an intellectual disability who engage in the community groups, and the mentors and managers of the community groups in both sites exploring the facilitating factors and barriers to the adoption of the Matilda intervention.

Increasingly, qualitative, or mixed methods are being used in process evaluations of feasibility studies to capture salient information to inform a future randomised trial [46]. Quantitative methods will be used to assess recruitment rates, the numbers of adults with an intellectual disability and mentors matched, mentors’ weekly logs and fidelity checks. The dosage and intensity of the persons’ engagement with their local community group will also be analysed as part of the progression criteria.

### Process evaluation methodology

O’Cathain et al. [47, 48] identified four key areas that qualitative research can explore in a feasibility study: intervention, processes, outcomes, and measures. Their guidelines consist of 16 items within five domains: research questions, data collection, analysis, teamwork, and reporting. We will collect data on these five domains using focus groups involving 20 older adults with an intellectual disability (10 from Northern Ireland and 10 from London), their carers and mentors. We will explore the feasibility outcomes (recruitment, trial procedures, randomisation, mentors’ training, and supervision) and clinical outcomes (acceptability of measures, benefits, and challenges with delivering the Matilda project) and what needs modifying. We will ask some questions to the participants during the focus groups to determine how satisfied they were with the Matilda intervention and whether they would recommend it to a friend. We will also ask some questions about the appropriateness of the outcome measures, the ease of completion, and the time taken to complete them.

All participants will be invited to participate in the process evaluation. We will hold two focus groups of 6 to 8 adults with an intellectual disability who participated in the Matilda intervention in Northern Ireland and another focus group in London. There will be six focus groups in total with mentors, disability staff, and the managers of the local community groups in both sites. In addition, we will hold two focus groups with family/paid carers who participated in the Matilda intervention in both sites.

### Fidelity of intervention

We will collect information in the screening logs on how many eligible adults with an intellectual disability were randomised or the reasons for exclusion of those who were assessed for eligibility.

A checklist will be further developed to explore the fidelity of the Matilda intervention in greater detail in the process evaluation. In assessing the external validity, the extent of true collaboration in each local community group initiative will be assessed with the adults with an intellectual disability, carers, and mentors. To determine whether the Matilda intervention will be delivered as intended (adherence), each mentor will be asked to complete their own weekly paper checklist, which details attendance, frequency, activities, adverse events, etc.

We will measure the frequency of the delivery of the Matilda intervention monthly through the logs, process evaluation, and acknowledge the variability of the groups. Where possible, all the adults with an intellectual disability allocated to the intervention arm will be afforded the opportunity to access a group for the full 6-months. There may be occasions where some adults with an intellectual disability do not access their group, and this will be explored and recorded.

### Quantitative data analysis

We will use descriptive statistics to analyse the feasibility outcomes; these will include the distribution of scores, mean, standard deviation, medians, frequencies, and percentages. Qualitative and quantitative data will be triangulated to validate their findings and provide a more in-depth understanding regarding what is observed.

be measured using a combination of descriptive and inferential statistics. Eligibility assessment will be quantified by calculating the proportion of screened individuals who meet inclusion criteria. Recruitment rates (number of eligible participants screened divided by number of participants consented) and pathways will be analysed using frequency distributions to determine the most effective channels for enrolment of the older adults with intellectual disability. The consent rate will be measured as the percentage of eligible adults with intellectual disability who agree to participate. The overall retention rate will be calculated as the proportion of older adults with intellectual disability completing the study. These measures collectively inform the feasibility of implementing and scaling the Matilda intervention effectively for a definitive RCT [49, 50].

### Qualitative data analysis

These focus groups will be audio recorded and transcribed, stored via NVivo software and analysed using the 6-stage process by Braun and Clarke [51] a deductive thematic analysis. To ensure the quality of the data and trial, we will establish an independent Trial Steering Committee (TSC) and an independent Data Monitoring Ethics Committee (DMEC), which consists of experts outside this feasibility study.

### Health economics evaluation

The economic evaluation will explore issues likely to be encountered in the conduct of a full economic appraisal, including sources of data, how best to collect these, and the inclusion of spill-over effects into the analysis. We will assess the feasibility of calculating quality adjusted life years (QALYs) using both the EQ5DY and WHOQOL-DIS and including spill-over effects experienced by mentors and carers in the evaluation.

Costs of providing the intervention (including recruitment, training, and expenses related to the supervision by the project manager) will be collected from the project manager in collaboration with participating sites. Information on resource use will be collected using an adapted version of the Client Service Receipt Inventory. Data will be collected at baseline, 6 and 12 months post-intervention. Resource utilization will cover the period since the last data collection point at 6 and 12 months: and in the previous 6 months in the case of baseline measurement. Health and social care use will include contacts with health professionals such as GPs, psychiatrists, psychologists, community nurses, social workers, and community intellectual disability teams, as well as use of hospital accident and emergency, inpatient, and outpatient services. Information on prescription of medicines will also be collected.

Resource use will be monetised using published sources, PSSRU [52], NHS reference costs [53] and the British National Formulary [54]. Costs will be reported from a health and social care perspective. In the base case analysis, we will focus on costs and outcomes as they accrue to the participant with an intellectual disability only. Total costs in each randomised group will be compared using a Generalised Linear Regression Model as costs are likely to be non-zero and positively skewed.

We will explore estimation of the incremental mean cost per QALY gained from the intervention compared to the control group and the cost-effectiveness of the intervention across other outcome measures. If calculable, the mean QALY per participant with an intellectual disability will be calculated as the area under the curve for the duration of the trial, adjusting for baseline values. CIs will be constructed using non-parametric bootstrapping with replacement. This exercise will be explored for outcome measures generated using EQ5D if possible and cost-effectiveness using WHOQOL-DISF.

## Discussion

In partnership with our PPI organisation, CAN Advocacy Support Network, we have seen strong enthusiasm from older adults with intellectual disabilities who value opportunities to join local community groups and form new friendships. Likewise, community organisations have expressed willingness to welcome and support inclusion. The MATILDA feasibility trial will build on this momentum, extending earlier work to understand how community networks can be strengthened and what practical supports are needed for mentors and groups to sustain engagement.

Beyond testing feasibility, MATILDA contributes to the broader implementation science agenda by examining how inclusive community models can be embedded within existing infrastructures and sustained over time. Using implementation frameworks will help identify barriers and enablers to adoption, assess readiness for change, and generate learning relevant to future commissioning and policy reform. In doing so, MATILDA aligns closely with the NIHR Implementation Science priorities and UK policy directives such as NICE NG96 and the Adult Social Care Reform White Paper, which advocate for scalable, community-based approaches that promote independence, inclusion, and wellbeing.

However, feasibility challenges are anticipated. Recruitment may be constrained by the availability of suitable mentors and the capacity of services to identify eligible participants. The sample, drawn from specific UK contexts, may limit generalisability. In addition, the evolving post-pandemic environment continues to affect community participation and volunteer engagement. Addressing these challenges will be critical to refining recruitment pathways, support structures, and intervention delivery ahead of a definitive trial.

## Trial status

Protocol version 5.0, 28/12/2023. Recruitment started in June 2023 and completed in March 2024. Data collection commenced in June 2023 and will continue through to June 2025.

## Supplementary Information

Supplementary Material 1.

## Data Availability

Not applicable.

## Abbreviations

MATILDA: Managing Activities Together, to Involve older adults with a Learning Disability in their local community
PIS: Participant Information Sheet
PPI: Patient and Public Involvement
RA: Research Associate
RCT: Randomised controlled trial
TTR: Transition To Retirement
